# Serum irisin level, insulin resistance, and lipid profiles in patients with hidradenitis suppurativa: a case-control study^⋆^^⋆⋆^

**DOI:** 10.1016/j.abd.2020.04.009

**Published:** 2020-09-11

**Authors:** Ezgi Özkur, Yasemin Erdem, İlknur Kıvanç Altunay, Damla Demir, Nurcihan Çalışkan Dolu, Erdinç Serin, Aslı Aksu Çerman

**Affiliations:** a Department of Dermatology, University of Health Sciences, Şişli Etfal Training and Research Hospital, İstanbul, Turkey; b Department of Biochemistry, University of Health Sciences, Şişli Etfal Training and Research Hospital, İstanbul, Turkey

**Keywords:** Hidradenitis suppurativa, Insulin, Metabolic diseases

## Abstract

**Background:**

Hidradenitis suppurativa is a chronic inflammatory skin disease of terminal follicular acroinfundibulum.

**Objectives:**

This study aimed to evaluate serum irisin, plasma glucose, insulin, and lipid levels in hidradenitis suppurativa, and elucidate possible associations with disease activity, inflammatory, or metabolic parameters.

**Methods:**

This case-control study included 37 patients (M/F: 9/28) and 37 sex-, age- and body mass index -matched healthy controls (M/F: 11/26). Demographic data, Hurley stage of disease, fasting glucose, insulin, total cholesterol, high density lipoprotein cholesterol, low-density lipoprotein cholesterol, triglycerides, C-reactive protein levels, erythrocyte sedimentation rate, hematologic parameters, and serum irisin were assessed.

**Results:**

The hidradenitis suppurativa group had significantly higher waist circumference than controls (*p* < 0.001). Insulin resistance, defined as a homeostatic model assessment for insulin resistance value greater than 2.5, was observed in 45.9% of patients and 8.1% of controls (*p* = 0.003), whereas metabolic syndrome was observed in 32.4% of patients and 5.4% of controls (*p* < 0.001). Furthermore, plasma triglycerids, glucose, and insulin levels were significantly higher in the hidradenitis suppurativa (*p* = 0.013, *p* = 0.001, and *p* = 0.004), respectively. Mean irisin level was insignificantly higher in the hidradenitis suppurativa group (37.4 ± 32.6) than in controls (26.2 ± 24.7, *p* = 0.217).

**Study limitation:**

Physical activity and the exercise levels of participants were not documented.

**Conclusion:**

This study indicates that hidradenitis suppurativa patients have higher serum irisin, fasting plasma glucose, insulin, and triglycerides levels than healthy controls. Thus, the authors suggest that hidradenitis suppurativa patients should be evaluated for insulin resistance and metabolic syndrome, and monitored accordingly.

## Introduction

Hidradenitis suppurativa (HS) is a chronic, inflammatory skin disease that is believed to begin with disturbed keratinization of the follicular infundibulum and results from the occlusion of the terminal follicular acroinfundibulum, leading to relapsing painful inflammatory nodules, abscesses, fistulas, and scarring. It primarily affects intertriginous, apocrine gland-bearing areas, most commonly in the axillae, inguinal and anogenital regions.^1^, ^2^ The prevalence of HS reported in literature ranges between 0.2% and 4%.^3^ One study, conducted with 1776 HS patients, observed that dyslipidemia, obesity, metabolic syndrome (MS), hypertension, diabetes mellitus (DM), thyroid disease, polycystic ovarian syndrome (PCOS), and psychiatric disorders occurred at increased rates in patients with HS.^4^ Furthermore, Egeberg et al., reported that HS was associated with a significantly increased risk of myocardial infarction, ischemic stroke, and cardiovascular disease (CVD)-associated death.^5^ As a result, HS is starting to be recognized as a systemic inflammatory condition.^6^

Insulin resistance (IR) is a strong predictor of type 2 DM and also plays an important role in the risk of MS and dyslipidemia. It is known that the risk of atherosclerosis, CVD, and major adverse cardiovascular events is increased in patients with DM, MS, and dyslipidemia.^7^ Both adipose and muscle tissues secrete cytokines and other peptides, named adipokines and myokines, which regulates human metabolism by tissue communication, which is essential to maintain metabolic homeostasis. Akdoğan et al. found higher visfatin (an adipokine) levels in HS patients than in healthy age- and sex-matched controls.^8^ Irisin is a novel myokine, processed from the product of the fibronectin type III domain-containing protein 5 (FNDC5) gene prior to being released into the circulation and regulated by peroxisome proliferator-activated receptor gamma coactivator 1-alpha (PGC-1α).^9^ This myokine is mainly secreted from skeletal muscle and the possible relationship among obesity and associated metabolic disturbances, including insulin sensitivity, was investigated in literature.^10^ Some authors assumed that irisin could be an indicator of body fat mass, as it was elevated in obese and MS subjects in many studies.^11^

This study aimed to assess serum irisin levels, lipid profiles, frequency of IR and MS in patients with HS, and compare them with healthy controls.

## Methods

The study included 37 patients (M/F: 9/28) and 37 sex, age, and body mass index (BMI)-matched healthy controls (M/F: 11/26) that met the inclusion criteria: age ≥18 years, a negative history of cancer, and HS diagnosis confirmed by dermatologists, according to the Dessau definition.^2^ Exclusion criteria were pregnancy, lactation, active or chronic infection, having another dermatological disease, or having a prior metabolic disease (thyroid disorders, DM, MS, PCOS, and Cushing syndrome, among others) or autoimmune or connective tissue diseases, documented history of major adverse cardiovascular events, chronic liver or kidney disease and current use of antihyperlipidemic agents, antidiabetic treatments, or other agents that might affect carbohydrate metabolism (e.g.: corticosteroids, retinoids). None of the participants were under any dietary restriction or systemic treatment for HS for at least six months prior to enrollment to study. Those who engaged in professional sport practice were excluded. All participants gave signed an informed consent before their inclusion in the study, and the study was approved by the Ethical Committee.

Demographic data, including age, gender, cigarette smoking status, daily alcohol consumption, disease duration, and disease severity were recorded. Disease severity was evaluated at the time of clinical examination with Hurley staging. Waist circumference (WC; cm), height (m), weight (kg), systolic blood pressure (BP) diastolic BP, and BMI (kg/m^2^) were recorded for all patients and controls.

Blood samples of peripheral venous blood were drawn after an overnight fast in all participants, and serum total cholesterol (TC), high density lipoprotein cholesterol (HDL-c), low-density lipoprotein cholesterol (LDL-c), triglycerides, glucose, insulin, C-reactive protein (CRP) levels, erythrocyte sedimentation rate (ESR), and hematologic parameters were assessed. For analyzing irisin, blood samples were centrifuged for 15 min at 1000 *g*, then serum was stored at −80 °C. Irisin serum levels were measured by enzyme-linked immunosorbent assay (ELISA) supplied by ElabScience Kit (Wuhan, China, Catolog no. E-EL-H2254) with assay range: 0.16–10 ng/mL and a sensitivity of 0.10 ng/mL. Samples were assessed with 1/10 dilution with an intra-assay coefficient of variation of 4.8% and an inter-assay coefficient of variation of 5.6%.

MS was diagnosed by the presence of three or more criteria according to the National Cholesterol Education Program's Adult Treatment Panel III (ATP-III): WC > 102 cm in men or >88 cm in women; hypertriglyceridemia ≥ 150 mg/dL; HDL-c < 40 mg/dL in men and <50 mg/dL in women; BP ≥ 130/85 mmHg or current use of medication for hypertension; fasting plasma glucose ≥ 110 mg/dL. The homeostatic model assessment of insulin resistance (HOMA-IR) was calculated using this formula: fasting insulin level (μIU/mL) × fasting glucose level (mg/dL)/405. IR was defined as HOMA-IR > 2.5.^12^, ^13^

Data were analyzed using IBM SPSS 15.0 for Windows v.21.0. (IBM Corp., Armonk, NY). Descriptive statistics were given as numbers and percentages for categorical variables, and means and standard deviations for numeric variables when appropriate. When parametric assumptions were met, the independent Student's *t*-test was used to compare numeric variables between patients and controls, and the Mann–Whitney's *U* test was used to compare numeric variables when parametric assumptions were not met. The chi-squared test was used to compare between group differences in categorical variables. Spearman's correlation coefficient was used to analyze the association between numerical variables. The level of significance level was accepted as *p* < 0.05.

## Results

Table 1 shows the main demographic and clinical findings of 37 patients and 37 controls. Patients and age, sex, BMI-matched control group were similar in height, weight, smoking habits, and excessive alcohol consumption. Mean disease duration was 85.6 ± 88.9 months. Although BMIs were similar in groups (*p* = 0.068), the HS group had significantly higher WC than healthy controls (*p* < 0.001). IR was observed in 45.9% of patients and 8.1% of controls (*p* = 0.003) whereas metabolic syndrome was observed in 32.4% of patients and 5.4% of controls (*p* < 0.001). Eight HS patients (21.6%) were classified as Hurley stage I, 14 (37.8%) as stage II and 15 (40.5%) as stage III.

**Table 1:** Demographic and clinical findings of patients with hidradenitis suppurativa and healthy controls

		HS patients (*n* = 37)		Controls (*n* = 37)		*p*
		Mean ± SD	Min–max (median)	Mean ± SD	Min–max (median)	
Age (years)		34.6 ± 10.9	17–62 (34)	36.2 ± 9.5	18–51 (35)	0.423
Sex *n* (%)	Female	9 (24.3)		11 (29.7)		0.601
	Male	28 (75.7)		26 (70.3)		
Height (cm)		170.9 ± 8.6	150–191 (170)	172.5 ± 7.5	157–185 (172)	0.341
Weight (kg)		85.8 ± 20.6	40–130 (82)	79.2 ± 18.8	49–110 (80)	0.161
BMI (kg/m^2^)		29.3 ± 6.4	16–42.4 (27.9)	27.1 ± 5.0	21.3–36.1 (25.1)	0.068
Disease duration (months)		85.6 ± 88.9				
Current smoker *n* (%)		20 (54.1)		15 (40.5)		0.244
Excessive alcohol consumption n (%) (>2 doses per day)		10 (27.0)		11 (29.7)		0.797
Systolic blood pressure (mmHg)		116.6 ± 12.7	90–150 (120)	115.4 ± 9.0	100–130 (120)	0.612
Diastolic blood pressure (mmHg)		67.7 ± 16.3	10–95 (70)	65.0 ± 8.0	40–80 (60)	0.070
Waist circumference (cm)		98.8 ± 19.1	50–145 (98)	79.9 ± 14.0	60–105 (80)	<0.001
Metabolic syndrome *n* (%)		12 (32.4)		2 (5.4)		0.003
Insulin resistance *n* (%) (HOMA-IR value of >2.5)		17 (45.9)		3 (8.1)		<0.001
Hurley stage *n* (%)	1	8 (21.6)				
	2	14 (37.8)				
	3	15 (40.5)				

BMI, body mass index; HOMA-IR, homeostatic model assessment for insulin resistance.

Triglycerids, plasma glucose, and insulin levels were significantly higher in HS group and there was no significant statistical difference in terms of TC, HDL-c, and LDL-c between patients and controls (Table 2). The mean values of irisin were 37.4 ± 32.6 in patients and 26.2 ± 24.7 in controls, but this difference was not statistically significant (*p* = 0.217). CRP, ESR, leukocyte, and neutrophil count were significantly higher in patients with HS. WC was positively correlated with irisin both in the patient (*r* = 0.495, *p* = 0.002) and control (*r* = 0.484, *p* = 0.002) groups. BMI had a positive correlation with irisin only in the control groups (*r* = 0.523, *p* = 0.002). HDL-c was negatively correlated with irisin (*r* = −0.495, *p* = 0.005); in turn, LDL-c was positively correlated with irisin (*r* = 0.327, *p* = 0.048) in control group. Spearman correlation analysis results showed no further correlation between irisin and other investigated parameters.

**Table 2:** Comparison of laboratory parameters in patients with hidradenitis suppurativa and healthy controls

	HS patients (*n* = 37)		Controls (*n* = 37)		*p*
	Mean ± SD	Min–max (median)	Mean ± SD	Min–max (median)	
TC **(**mg/dL)	192.8 ± 42.3	104–298 (192)	186.1 ± 37.8	115–290 (184)	0.489
HDL-c (mg/dL)	47.5 ± 16.2	26–126 (45)	46.1 ± 10.9	27–71 (44)	0.991
LDL-c (mg/dL)	112.8 ± 33.9	11–178 (117)	122.3 ± 28.0	69–190 (121)	0.420
Triglycerides (mg/dL)	171.8 ± 147.6	50 − 886 (140)	102.8 ± 49.0	30–244 (102)	0.013
Fasting plasma glucose (mg/dL)	99.5 ± 14.1	76–142 (98)	89.8 ± 8.5	75–116 (89)	0.001
Fasting plasma insulin (μIU/mL)	11.6 ± 8.8	3.1–56.1 (10)	8.1 ± 3.0	3.5–21.5 (8.1)	0.004
HOMA − IR	2.93 ± 2.65	0.73–16.8 (2.48)	1.8 ± 0.74	0.77–5.11 (1.86)	0.001
Irisin (ng/mL)	37.4 ± 32.6	1.05–121.7 (34.2)	26.2 ± 24.7	1.8–113.9 (19.4)	0.217
CRP (mg/dL)	8.0 ± 8.6	0.1–39.9 (5.5)	5.9 ± 16.6	0–101.5 (2.4)	0.002
ESR (mm/h)	17.1 ± 14.7	2–76 (12)	12.5 ± 13.0	2–55 (7.5)	0.021
Platelet (10^6^/mm^3^)	286.1 ± 72.2	138–488 (277)	272.2 ± 60.7	165–465 (254)	0.312
Hemoglobin (g/dL)	14.1 ± 1.9	9.7–16.5 (14.6)	14.4 ± 1.5	10.4–17 (14.7)	0.733
Leukocyte (10^9^/mm^3^)	8.9 ± 2.2	5.9–15.5 (8.6)	6.9 ± 1.5	4.2–10.2 (6.7)	<0.001
Neutrophil (10^9^/mm^3^)	8.8 ± 12.9	3.3–61.8 (4.8)	15.3 ± 48.6	2.1–288 (4.15)	0.012

TC, serum total cholesterol; HDL-c, high density lipoprotein cholesterol; LDL-c, low-density lipoprotein cholesterol; HOMA-IR, homeostatic model assessment for insulin resistance; CRP, C-reactive protein levels; ESR, erythrocyte sedimentation rate; MPV, mean platelet volume.

No significant differences were observed in irisin and HOMA-IR values in comparisons according to Hurley stages in patients with HS (Table 3).

**Table 3:** Comparison of mean values of homeostatic model assessment of insulin resistance and irisin level in patients with hidradenitis suppurativa regarding Hurley stage

	Hurley stage						*p*
	1		2		3		
	Mean ± SD	Min–max (median)	Mean ± SD	Min–max (median)	Mean ± SD	Min–max (median)	
HOMA-IR	2.51 ± 2.24	0.73–7.88 (1.69)	2.48 ± 0.91	1.05–4.25 (2.36)	3.58 ± 3.74	1.13–16.77 (2.66)	0.241
Irisin level	34.9 ± 30.3	2.4–75.9 (37.7)	39.2 ± 42.4	1.1–121.7 (29)	36.9 ± 23.5	3.6–78 (37.4)	0.795

HOMA-IR, homeostatic model assessment for insulin resistance.

## Discussion

The present study demonstrated that patients with HS have a significantly higher IR and MS when compared with age-, sex-, BMI-matched control group. Moreover WC, triglycerids, fasting plasma glucose, and insulin levels were statistically higher in HS patients. In previous studies, HS patients were found to have higher BMIs than age- and sex-matched healthy controls. In the present study, the authors matched BMIs between two groups to eliminate potential confounders for metabolic homeostasis. Noteworthy, WC was still significantly higher in-patient group (*p* < 0.001). Previously, Miller et al. found similar results in WC, as well as increased BMI and waist-to-hip ratio in patients with HS when compared with controls.^14^ Furthermore, they found higher predicted basal metabolic rate for the HS groups when compared with healthy controls, and implied that increased basal metabolic rate in HS patients may reflect a dysfunctional metabolism contributing to the high body fat. Moreover, it is known that cardiovascular risk is higher in obese patients, especially in those with the abdominal/visceral adipose phenotype.^15^ Therefore, the present findings may indicate that HS patients are at higher risk of CV events.

In line with the present findings, Vilanova et al. included 76 patients with HS and 61 healthy controls and reported the prevalence of MS was three times more common in HS patients than in control subjects. In a multivariate analysis, after adjusting for age, sex, and BMI, those authors found that HS is a significant risk factor for IR.^16^ In this regard, Vita et al. claimed that dysregulation of mTORC1 signaling is the key of increased IR in HS: mTORC1 activates S6 kinase (S6K), and S6K causes phosphorylation and degradation of insulin receptor substrate 1/2, thus impairing insulin signaling.^17^ Another possible explanation for the association between HS and IR might be the presence of chronic inflammation due to pro-inflammatory cytokines such as TNF-α. Thus, overproduction of TNF-α may impair insulin signaling through inhibition of tyrosine kinase activity of the insulin receptor, suppressing the secretion of adiponectin from adipocytes. Adiponectin is known as an anti-inflammatory molecule that also regulates insulin sensitivity. Malara et al. found that HS patients have decreased adiponectin levels than healthy age, sex matched controls.^18^

In the present study, MS (defined in accordance with the ATPIII criteria) was more frequently observed in HS patients than in the control group. HS has been associated with MS and obesity in previous studies.^19^ Not surprisingly, the risk of CVD-associated death was higher in patients with HS compared with controls in previous studies.^20^ Kohorst et al. concluded that this risk was higher than that observed in psoriatic patients.^6^ Moreover, Gonzalez-Lopez et al. noted that there is a premature and accelerated development of atherosclerosis in patients with HS, suggesting that HS itself may be an independent risk factor for early atherosclerotic cardiovascular disease and related major adverse cardiovascular events.^21^ It was reported that the prevalence of dyslipidemia was higher in HS patients than in the normal population.^22^ In the present study, a statistically significant difference was observed only in plasma triglyceride levels, not in TC or LDL-c levels in patients with HS.

Irisin is a myokine that leads to increased energy expenditure due to its ability to stimulate the “browning” of white adipose tissue.^23^ In the present study, plasma irisin levels were higher in HS patients than controls; however, this difference was not statistically significant (*p* = 0.217). Huh et al. reported that diabetic patients had higher levels of irisin and stated that irisin was an independent predictor of DM.^24^ In another study, women with PCOS who were overweight or obese had elevated levels of irisin (∼15%–20%) when compared with controls of normal weight subjects.^25^ Similarly, it was reported that weight loss (−6.31 ± 0.195%) leads to a significant decrease in circulating irisin (15%), and weight regain causes irisin levels to return to baseline.^26^ Crujerias et al. claimed that, in healthy individuals, most of the irisin is produced in muscle cells, but, in obesity, the amount of irisin secreted from adipose tissue is probably higher.^27^ In present study, the patient and control groups were BMI-matched. Therefore, this difference in irisin levels cannot be attributed to increased adipose tissue. Furthermore, studies showed that there is a positive correlation between irisin and HOMA-IR.^28^, ^29^ In line with this, the present HS patients may have higher levels of irisin due to higher HOMA-IR values, but this correlation was statistically insignificant (*r* = 0.036, *p* = 0.836). Moreover, irisin levels are found to be positively associated with the risk of the MS in white individuals (OR = 9.44).^30^

Increased irisin levels were associated with major adverse CV events in patients with established coronary artery disease after percutaneous coronary intervention.^31^ In a recent study, Baran et al.^32^ found that serum irisin levels were insignificantly increased in psoriatic patients when compared with healthy controls (*p* = 0.38). They also observed a positive statistical relation with irisin and lipocalin-2 (LCN-2).^33^ LCN-2 has been associated with IR, DM and obesity-induced entothelial dysfunction. Baran et al. also have reported that serum LCN-2 was significantly overexpressed in psorasis patients when compared with healthy individuals. Interestingly, Wolk et al. found strongly elevated LCN-2 expression in HS lesions, and those authors claimed that granulocytes and keratinocytes were the sources of this expression. They suggested that LCN-2 might serve as a blood biomarker for the objective assessment of inflammatory activity in HS.^34^ In a later research, those authors reported that the prevalence of HS was increased in patients with psoriasis.^35^ The similar results of studies with irisin and LCN-2 in HS and psoriasis patients may indicate a possible pathogenetic link between these two conditions.

The strength of the present study is that it was the first to evaluate irisin in HS and its relations with IR and other metabolic parameters. However, there are some limitations. First, a small sample was assessed. Second, although subjects who engage in professional sports practice were not enrolled (as irisin is a myokine), physical activity and the exercise level of the patients and controls were not documented.

## Conclusion

Patients with HS have higher fasting plasma glucose, insulin, triglyceride, and serum irisin levels than controls. An increased frequency of IR and MS was observed in HS. The results of the present study call for increased awareness of the association between HS and dysregulated metabolic homeostasis; physicians should be aware of its clinical consequences and overall comorbidity burden in patients with HS.

## Financial support

None declared.

## Authors’ contributions

Ezgi A-zkur: Statistical analysis; approval of the final version of the manuscript; design and planning of the study; drafting and editing of the manuscript; collection, analysis, and interpretation of data; effective participation in research orientation; intellectual participation in the propaedeutic and/or therapeutic conduct of the studied cases; critical review of the literature; critical review of the manuscript.

Yasemin Erdem: Approval of the final version of the manuscript; drafting and editing of the manuscript.

İlknur Kıvanç Altunay: Critical review of the literature.

Damla Demir: Collection, analysis, and interpretation of data.

Nurcihan Çalışkan Dolu: Collection, analysis, and interpretation of data.

Erdinç Serin: Critical review of the literature.

Aslı Aksu Çerman: Design and planning of the study; effective participation in research orientation.

## Conflicts of interest

None declared.
